# The Yellow Fever Epidemic at Columbus, Texas, October, 1873

**Published:** 1903-11

**Authors:** 


### The Yellow Fever Epidemic at Columbus, Texas, October, 1873. Letter from Dr. J. M. Bowers, of Columbus, to Dr. C. O. Weller, of Austin.

Dr. Weller has kindly placed Dr. Bowers' letter at my disposal, with Dr. Bowers' permission to publish it. As it bears on the subject now foremost in the minds of the profession of Texas, and seems to show that the mosquito was not the factor in the introduction and spread of the fever at Columbus, it will be read with interest. Second and numerous cases occurred after exposure to first case—the Swede—in less time than it is possible for the mosquito to "ripen" and inoculate a person and then the period of incubation (seventeen days in all, I believe it is agreed, is the minimum.) It seems, further, to prove that there is some connection between yellow fever and dengue, as dengue was prevailing in Columbus at the time the Swede came into town from Shreveport, where yellow fever was then epidemic. There is a discrepancy in Dr. Bowers' dates. He says that he thinks it was October 10th when the man died. Further on he says that he went to Tom Oakes', near Glidden, the morning the Swede died, and returned on October 18th. As Glidden is only a few miles away, and Dr. Bowers returned in a few hours, the latter date must be correct. It coincides also, or nearly so, with Dr. Weller's recollection. Dr. Weller says the Swede died on October 15th, and that he (Dr. Weller), was attacked on 19th. Dr. Bowers adds: "That night (18th) I went to see Sachs, and the fever was epidemic."

So that we see the infection introduced into a city not earlier than 12th, and the fever raging as an epidemic by 18th, six days.

Columbus, Texas, October 28, 1903.

*D. C. O. Weller, Austin, Texas.*

Dear Doctor: Your letter to hand, in which you ask me about the yellow fever epidemic at this place in 1873. Not being able to write on account of cataract in both eyes, I am compelled to dictate you this letter. So much for that.

The man who introduced the yellow fever here came from Shreveport, where the fever was prevailing at the time. He stopped at McDonald's boarding house, which was located just south of the court house, and went to your drug store and bought a dose of salts, returned to his boarding place, took to his bed and never left it again. He was for six or seven days without physician or attention. I attended a railroad man in the next bed to him, who had malarial fever, and who got well. When I discharged him the boarding house keeper asked me to see this man, who, by the way, was a Swede. I did so, and found his bed full of black vomit; the color of his skin, a dirty yellow; blood oozing from his nose and gums, and evidently nearing his death. He had all the symptoms of yellow fever—a disease with which I was well acquainted, having been through epidemics at New Orleans, Houston and Matamoros, and I could not easily be mistaken about his case. Upon leaving the case, some of the leading citizens came and asked me about it, and I told them it was yellow fever. They told me that I was bound to be mistaken, and that I was injuring the town. I told them that I did not make the yellow fever or bring it to town, but that the case was undoubtedly yellow fever. The man died in about two hours after I saw him, and was buried immediately. I think this was the tenth day of October, 1873, and he had been here five or six days. On the day of his death I was called out of town to see Tom Oakes, who was dangerously sick, and remained with him until about dark on Saturday, the 17th. About two hours before day, of the 18th, you came to my house and called me up and wanted me to go and see some cases you then hand on hand. The first place you carried me to was to see a man named Sachs, near where J. N. Binkley then lived, in the south part of the town. Upon our arrival at the house we found the man had just died. There was a big bowl of black vomit sitting by his bedside. I took the light and looked at him closely. I found his gums spongy and bleeding, and the color of his skin a dirty yellow. I remember filling a bottle with the black vomit and putting it into my pocket, to be examined at my leisure. From this house you then carried me to the passenger depot—one room of which was occupied by the telegraph operator—to see the operator, who was named Russell, I believe. We found him sick in bed. I examined him very closely, and upon leaving pronounced it a case of decided and fatal yellow fever. Russell died before noon of that day, which was Sunday, October 18, 1903. You asked me what was to be done, and I replied, proclaim it epidemic, as it was in both ends of town, so that the people could leave. Which they did. About eight or nine o'clock that morning Dr. Jo Brown requested me to go with him to see some of his cases. He carried me to McDonald's boarding house—where first case, that of the Swede, had died—where I found six men down with yellow fever, being all of the boarders at the house, all of whom died before the night of the next day; that is, the night of the 19th. One young man, Lacy, had left the house and gone to his grandfather's house, in another part of the town, where he took the fever and died, as did also his grandmother. From this time the fever spread rapidly all over town, and killed some of our best citizens. You and W. T. Burford were among the earlier cases, and both recovered. Negroes were singularly exempt from the disease, there being but four deaths among them—all mulattoes. The last two cases occurred on Christmas day, a Mr. Shuttlewurse and mulatto boy, both of whom died on the thirty-first day of December, 1873. Many people took the disease after they had fled the town, and some eight or ten deaths occurred among these, but in no instance was the fever communicated to those with whom they stayed. The total number of deaths was one hundred and one.

I am sorry that I can not give you a better and clearer account of the epidemic, but it has now been a long time ago. I remain,

Your friend,

JOHN M. BOWERS.

Dr. Weller wrote to Dr. Bowers, asking if he were not mistaken as to date of the several deaths. The following is Dr. Bowers' reply:

COLUMBUS, TEXAS, November 5, 1903.

*C. O. Weller, M. D., Austin, Texas.*

MY DEAR DOCTOR: As a matter of course an old man, 86 years old, has not as good memory as yours, but I think you are mistaken about the Swede's death. The morning he died I went to Tom Oakes', near Glidden, and returned on the eighteenth of October, 1873. That night I went to see Sachs, and the fever was epidemic.

Tell Dr. Daniel I have no objection to his publishing any part of my letter. As a matter of course it was not written for publication, or it would have been written better.

Your friend forever,

JOHN M. BOWERS.

